# Genomics Insights into the Demographic History and Introgression of Tibetan Pigs

**DOI:** 10.3390/ani16091328

**Published:** 2026-04-27

**Authors:** Pengxiang Xue, Chengwan Zha, Yabiao Luo, Ning Huang, Nian Liu, Hao Wang, Meiying Fang

**Affiliations:** State Key Laboratory of Animal Biotech Breeding, MOA Key Laboratory of Animal Genetics and Breeding, College of Animal Science and Technology, Frontiers Science Center for Molecular Design Breeding, Beijing Key Laboratory for Animal Genetic Improvement, China Agricultural University, Beijing 100193, China; xuepengxiang98@163.com (P.X.); cwzha@cau.edu.cn (C.Z.); yabiaoluo@cau.edu.cn (Y.L.); 15390751795@163.com (N.H.); nianlauu@163.com (N.L.); wanghao0051@163.com (H.W.)

**Keywords:** Tibetan pigs, demographic history, introgression, adaptation

## Abstract

Tibetan pigs are an important indigenous genetic resource on the Qinghai-Tibetan Plateau, but their population history remains unclear. Here, we analyzed whole-genome resequencing data from 29 Tibetan pigs and 69 wild boars from different regions to investigate their genetic structure and origin. Population structure analyses showed that Tibetan pigs formed a distinct genetic cluster, but clear substructure was present among the four Tibetan pig groups. Demographic analyses indicated that all Tibetan pigs shared a similar deep ancestral background, whereas the Tibetan population from Tibet (TT) showed a distinct recent demographic trajectory relative to the other Tibetan pig groups. Model inference based on multidimensional site frequency spectra further supported a mixed origin of TT from northern and southern Asian wild boar-related ancestral components. In contrast, the other three Tibetan pig groups were best explained as lineages derived from TT after its formation. We also detected a persistent western Eurasian-related excess-sharing signal in TT, consistent with potential introgression. Selection scans identified candidate genes and pathways potentially related to hypoxia adaptation, cardiovascular function, and lung development.

## Simple Summary

In this study, we analyzed high-depth whole-genome resequencing data from 69 wild boars from 17 geographic regions and 29 Tibetan pigs from four regions to investigate the demographic history of different Tibetan pig groups. Our results showed that Tibetan pigs from Tibet had a demographic history distinct from that of the other three Tibetan pig groups. Demographic modelling based on the multidimensional site frequency spectrum indicated that Tibetan pigs from Tibet were best explained by a mixed origin involving both southern and northern wild boars, whereas the other three Tibetan pig groups were more likely to have diverged after the formation of the Tibetan pig population from Tibet. In addition, we detected a potential introgression signal between a wild boar from the Caucasus region and Tibetan pigs from Tibet, which may have contributed, at least in part, to adaptation to the high-altitude environment.

## 1. Introduction

The Qinghai-Tibetan Plateau is one of the most challenging environments for domestic animals because of chronic hypoxia, low temperature, and strong ultraviolet radiation [1,2]. Tibetan pigs are small-bodied, black-coated indigenous pigs distributed across high-altitude regions of the Qinghai-Tibetan Plateau and adjacent areas (Supplementary Figure S1). Tibetan pigs are one of the few indigenous pig populations that have long persisted under these conditions and are therefore an important model for studying high-altitude adaptation in livestock [3]. Previous physiological and genomic studies have shown that Tibetan pigs possess traits related to cardiopulmonary function, energy metabolism, and hypoxia response, and several candidate genes and pathways associated with altitude adaptation have already been reported [3,4,5]. Although Tibetan pigs are usually treated as a single high-altitude population, their origin is still debated. Earlier studies have suggested that Tibetan pigs are genetically close to lowland Chinese pigs [6,7]. However, population genetic analysis of Tibetan pigs from different regions shows that Tibetan pig populations are not genetically homogeneous, and there is a certain degree of genetic differentiation among Tibetan pigs in different regions [8,9].

Site frequency spectrum (SFS)-based demographic modelling has become a widely used framework for reconstructing population history from genomic data [10]. Compared with population structure analyses or phylogenetic trees alone, SFS-based approaches allow explicit testing of alternative demographic scenarios, including divergence, admixture, changes in effective population size, and secondary contact [11]. In particular, fastsimcoal2 estimates demographic parameters under complex evolutionary scenarios using a composite-likelihood framework based on the observed SFS, and has been widely applied to test divergence and admixture models [12]. Demographic inference from allele frequency spectra has been shown to be a powerful approach for evaluating alternative population histories in multiple populations.

In domestic animals, introgression is now recognized as an important evolutionary process that can contribute to environmental adaptation, local fitness, and phenotypic diversification [13]. In sheep, for example, adaptive introgression from wild relatives has been reported in Tibetan sheep, and broader population-genetic studies have also suggested historical admixture links between sheep from the Caucasus region and breeds from Tibet [14,15]. These examples show that gene flow from external lineages may leave biologically meaningful signals in high-altitude livestock.

In the present study, we analyzed whole-genome resequencing data from Tibetan pigs from four regions and wild boars from multiple regions to clarify the population structure, demographic history, and origin of Tibetan pigs. We first characterized the genetic structure of Tibetan pigs relative to Asian and western Eurasian wild boars. We then focused on the origin of Tibetan pigs from Tibet (TT) using demographic modelling. On this basis, we further tested whether the other three Tibetan pig groups were independently derived from northern or southern Asian wild boars, or instead originated after the formation of TT. Finally, we evaluated potential western Eurasian-related introgression in TT and screened genomic regions that may have contributed to high-altitude adaptation. The main aim of this study was to test whether the sampled Tibetan pig groups shared a common origin history or instead followed a hierarchical demographic framework. In this context, our main contribution is to show that Tibetan pigs are not genetically homogeneous and that the Tibetan pig population from Tibet occupies a central position in the demographic framework supported by the models tested here.

## 2. Materials and Methods

### 2.1. Samples

In this study, we analyzed high-depth whole-genome resequencing data from 100 individuals published in previous studies. These individuals included 2 *Sus cebifrons*, 69 wild boars from 17 geographic regions, and 29 Tibetan pigs from four regions, namely Tibetan pigs from Tibet (TT), Tibetan pigs from Gansu (GST), Tibetan pigs from Sichuan (SCT) and Tibetan pigs from Yunnan (YNT) (Supplementary Table S1). Sequencing depth exceeded 10× for all samples except for one wild boar individual from Armenia. Ethical review and approval were waived for this study due to no new data were generated.

### 2.2. Resequencing Reads Mapping and SNP Calling

Raw paired-end reads were first processed with fastp (v0.23.4) [16] for quality control. Clean reads were then aligned to the T2T_pig1.0 genome [17] using the MEM algorithm implemented in BWA (v0.7.19) [18]. The resulting alignments were sorted with SAMtools (v1.15) [19], and PCR duplicates were removed using Picard (v2.25.3). Variant calling was conducted with the HaplotypeCaller module in GATK (v4.2.5) [20]. Variant calling was performed separately for each chromosome to generate individual GVCF files. For each chromosome, GVCFs from all individuals were combined using CombineGVCFs, followed by joint genotyping with GenotypeGVCFs. Chromosome-level variant files were then merged with MergeVcfs to obtain a genome-wide VCF dataset. To retain high-confidence variants, raw SNPs were filtered using the following criteria: QUAL < 30.0, QD < 2.0, MQ < 40.0, FS > 60.0, SOR > 3.0, MQRankSum < −12.5, and ReadPosRankSum < −8.0. Additional SNP filtering was performed with PLINK (v1.9) [21] using the parameters --geno 0.1 --mind 0.1 --maf 0.05. The SnpEff (v4.3) [22] was used for annotation after SNPs calling.

### 2.3. Population Structure Analysis

To minimize the effect of linkage disequilibrium (LD) in downstream analyses, SNPs were pruned in PLINK (v1.9) using the parameter --indep-pairwise 50 10 0.2. Based on the filtered dataset, a neighbor-joining (NJ) tree was reconstructed from the genetic distance matrix using VCF2Dis (v1.53) [23]. Principal component analysis (PCA) was performed in PLINK (v1.9), and population structure was inferred with ADMIXTURE (v1.3.0) [24]. Genome-wide nucleotide diversity (π) was estimated using VCFtools (v0.1.16) [25] in sliding windows of 50 kb with a 20 kb step size. To reduce the potential effect of unequal sample sizes among groups on LD decay analysis, we randomly selected six individuals from each group and calculated pairwise r^2^ values using PopLDdecay (v3.29) [26].

### 2.4. Demographic Analysis

To infer changes in historical effective population size, we applied the pairwise sequentially Markovian coalescent (PSMC) model to Tibetan pigs from four regions, using one individual from each region with sequencing depth greater than 20×. File format conversion was performed using SAMtools (v1.15), and historical effective population size trajectories over approximately the past one million years were then estimated with PSMC [27]. To assess uncertainty, 80 bootstrap replicates were generated for each PSMC analysis. We further inferred historical Ne using SMC++ (v1.15.2) [28]. Unlike PSMC, SMC++ does not require phased haplotypes and can incorporate genotype information from multiple individuals, making it more suitable for resolving recent demographic history. In brief, each chromosome was independently converted into SMC format using vcf2smc.py. Historical changes in effective population size were then reconstructed using smc++ estimate. Following previous studies, the mutation rate was set to 3.6 × 10^−9^ per site per generation, and the generation time was assumed to be 3 years [29].

### 2.5. D-Statistics Analysis

We used the filtered SNP dataset to perform D-statistics in order to test for potential gene flow among populations. In each test, different populations were assigned as P1, P2, and P3, with *S. cebifrons* used as the outgroup. The numbers of ABBA and BABA site patterns were calculated using Dtrios in Dsuite (v0.5) [30]. A result was considered statistically significant when the absolute value of the Z-score was greater than 3. A positive D value indicates excess allele sharing between P2 and P3, whereas a negative D value indicates excess allele sharing between P1 and P3. The configuration D (X, TT; wild boar, CEB) was used to compare whether each Tibetan pig group (X = GST, YNT, or SCT) shared relatively more alleles with a given wild boar group than TT did. The configuration D (X, IMW; WEW, CEB) was used to test whether the excess WEW-related affinity observed in TT could be explained through IMW.

### 2.6. Outgroup f_3_ Analysis

To further quantify genetic affinity between populations, we calculated outgroup *f*_3_ statistics using *S. cebifrons* as the outgroup. These analyses were performed with qp3Pop (v701) in AdmixTools [31]. Higher *f*_3_ values indicate a greater extent of allele sharing between the two tested populations.

### 2.7. Fastsimcoal2

The demographic history of Tibetan pigs was inferred using coalescent-based simulations implemented in fastsimcoal2 (v2.7) [12]. Following previous studies [32,33], autosomal SNPs used for demographic analyses were filtered according to the following criteria: (1) located in intergenic regions; (2) outside CpG islands “https://hgdownload.soe.ucsc.edu/hubs/GCF/000/003/025/GCF_000003025.6/bbi/ (accessed on 23 February 2026)”; (3) without missing genotypes across all samples; (4) spaced at least 2 kb apart; and (5) carrying confidently inferred ancestral alleles, with ancestral states inferred using *S. cebifrons*. The multidimensional unfolded SFS was generated using easySFS.py “https://github.com/isaacovercast/easySFS (accessed on 23 February 2026)”. To assess the robustness of demographic inference to ancestral-state assignment, folded SFS-based analyses were additionally performed. To evaluate the effect of sample sizes, additional fastsimcoal2 analyses were performed on randomly down sampled datasets (five individuals per group, except GST with four individuals). Alternative demographic models were then tested to identify the most likely population history. Model optimization was carried out in two stages: an initial 25 cycles (-l 25), followed by an additional 40 optimization cycles (-L 65). For each model, 100,000 coalescent simulations (-n 100,000) were performed, and 100 independent runs with random starting values were used to identify the parameter set with the highest likelihood. Model fit was evaluated based on the difference between the maximum observed likelihood and the maximum estimated likelihood across all runs and all tested models. To estimate confidence intervals for demographic parameters, we performed 100 bootstrap replicates under the best-fitting model, and each bootstrap replicate was independently optimized 100 times to retain the best run. The mutation rate was set to 3.6 × 10^−9^ per site per generation, and the generation time was assumed to be 3 years

### 2.8. TreeMix Analysis

All sites containing missing genotypes were removed using VCFtools (v0.1.16). The filtered dataset was then pruned for linkage disequilibrium using PLINK (v1.9) with the parameter --indep-pairwise 50 10 0.2, and converted into the input format required by TreeMix using the plink2treemix.py script. Next, 500 bootstrap replicates were generated for tree reconstruction. In each replicate, TreeMix (v1.13) [34] was run with a different random seed, and the tree was rooted using *S. cebifrons* as the outgroup. The resulting bootstrap trees were then summarized with the Consense program (v3.697) [35] in the PHYLIP package to generate a consensus tree. Finally, this consensus tree was used as a fixed topology constraint in TreeMix (v1.13) to further evaluate models with different numbers of migration edges. The optimal number of migration edges was additionally evaluated using OptM. Residual covariance heatmaps were used to assess model fit across alternative migration-edge settings.

### 2.9. Introgression Scan

To identify putative introgressed regions across the genome, we performed sliding-window analyses using the Dinvestigate module in Dsuite (v0.5), based on the statistic D (P1, P2; WEW, CEB). The analysis was conducted with parameter -w 100,50. Windows were then ranked according to f_d_ values, and the top 1% of windows were defined as candidate excess-sharing windows for downstream comparison and interpretation.

### 2.10. Selective Sweep Analysis

We combined Fst, π ratio, and XP-EHH to investigate candidate selective sweep regions in Tibetan pigs. First, Fst and π ratio were calculated using VCFtools (v0.1.16) with 50 Kb sliding window and a 20 Kb step size. For genomic regions exhibiting reduced diversity, we calculated the log_10_(π wild boars/π Tibetan pigs). XP-EHH was calculated by selscan. Then, the intersection of the top 5% windows from three methods was defined as candidate selective sweep regions. The GO (Gene Ontology) enrichment analysis was completed using KOBAS [36]. Quantitative trait locus (QTL) enrichment analysis was additionally performed for the selected regions, and FDR correction was applied to the QTL results.

### 2.11. Phylogenetic Topology Weighting Analysis

Autosomal VCF files containing the required individuals were extracted. Ancestral lineage sequences were inferred using sticcs (v0.0.5) “https://github.com/simonhmartin/sticcs (accessed on 11 April 2026)”, with *S. cebifrons* specified as the outgroup to determine derived alleles. Subtree topologies were then quantified using twisst2 (v0.0.5).

## 3. Results

### 3.1. Whole Genome Sequencing of Tibetan Pigs and Wild Boars

**Figure 1:**
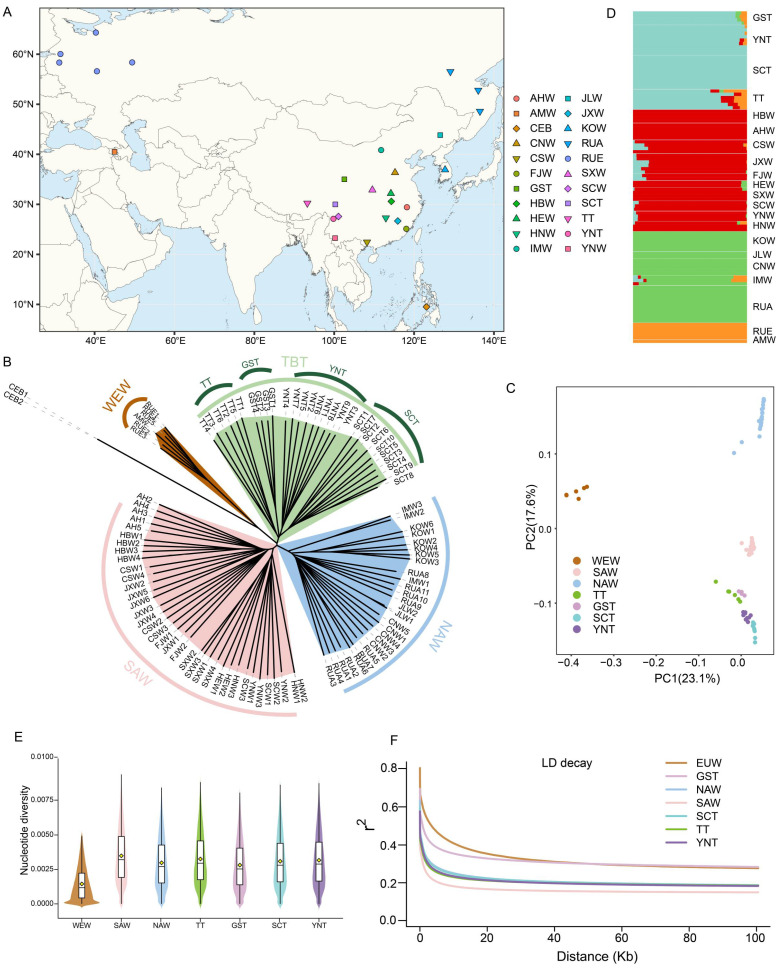
Population structure and genetic diversity patterns of Tibetan pigs and wild boars. (**A**) Geographic distribution of pig samples used in this study. (**B**) Neighbor-joining (NJ) tree constructed using genome-wide SNP data, with CEB used as the outgroup. (**C**) Principal component analysis (PCA) of all studied populations. (**D**) ADMIXTURE results for different ancestral components, with the lowest cross-validation error observed at K = 4. (**E**) Nucleotide diversity (π) of each population. (**F**) Linkage disequilibrium (LD) decay patterns of different populations. CEB, *Sus cebifrons*; WEW, western Eurasian wild boars; NAW, northern Asian wild boars; SAW, southern Asian wild boars; TBT, Tibetan pigs; TT, Tibetan pigs from Tibet; GST, Tibetan pigs from Gansu; YNT, Tibetan pigs from Yunnan; SCT, Tibetan pigs from Sichuan.

We aligned whole-genome resequencing data from two *S. cebifrons*, 69 wild boars from 17 regions, and 29 Tibetan pigs from four regions to the reference genome (Figure 1A and Supplementary Table S1). After quality control based on multiple criteria and filtering sites with MAF < 0.05 and max-missing > 0.1, a total of 31,108,496 high-quality SNPs were retained. The number of SNPs per individual ranged from 7,555,679 to 15,952,088 (Supplementary Table S1). After annotation using SnpEff, we found that the largest proportion of SNPs was located in intronic regions (21,773,061; 48.51%), followed by intergenic regions (18,369,676; 40.93%) (Supplementary Table S2).

### 3.2. Population Structure Analyses of Tibetan Pigs and Wild Boars

We further explored the population genetic structure of wild boars and Tibetan pigs. The neighbor-joining (NJ) tree revealed clear genetic differentiation among all samples (Figure 1B). Using *S. cebifrons* as the outgroup, all samples were divided into four major branches. The western Eurasian wild boar group (WEW) was closest to the outgroup, and RUE and AMW were admixed within this branch rather than forming two clearly separated clusters. The Tibetan pig group (TBT) formed an independent branch and was further subdivided into four lineages corresponding to TT, GST, YNT and SCT. The remaining Asian wild boars were separated into two major branches, namely northern Asian wild boars (NAW) and southern Asian wild boars (SAW). Overall, the NJ tree indicated that Tibetan pigs constituted a distinct genetic cluster, while substantial differentiation was also present among the four Tibetan pig groups. The principal component analysis (PCA) further supported the population structure inferred from the NJ tree (Figure 1C). PC1 primarily separated WEW from Tibetan pigs and Asian wild boars, reflecting the marked genetic divergence between western Eurasian wild boars and Asian pig populations. PC2 further distinguished NAW, SAW, and Tibetan pigs, and within the Tibetan pig group, TT and GST were positioned close to each other, whereas YNT and SCT also clustered together. The ADMIXTURE analysis was largely consistent with the NJ tree and PCA results (Figure 1D). Among the tested K values, the lowest cross-validation error was observed at K = 4 (Supplementary Figure S2). The samples were mainly assigned to four ancestry components corresponding to WEW, NAW, SAW, and TBT, which agreed with the overall pattern identified by the phylogenetic and PCA analyses. Notably, as K increased, TT consistently retained a WEW-related ancestry component, suggesting that the demographic history of TT may be more complex than that of the other Tibetan pigs (Supplementary Figure S3).

The analysis of nucleotide diversity (π) revealed substantial variation among groups (Figure 1E). Overall, WEW exhibited the lowest nucleotide diversity. Among Asian wild boars, NAW showed lower π values than SAW, indicating relatively reduced genetic diversity in the northern wild boar group. Within Tibetan pigs, GST displayed the lowest π value. The analysis of linkage disequilibrium (LD) decay also revealed marked differences among groups (Figure 1F). SAW showed the fastest LD decay, consistent with a relatively larger historical effective population size or weaker genome-wide linkage. In contrast, TT, SCT, YNT, and NAW exhibited highly similar LD decay patterns, all of which were slower than that observed in SAW. Among all groups, WEW showed the slowest LD decay, whereas within Tibetan pigs, GST exhibited the second slowest LD decay and showed substantially slower decay than the other three Tibetan pig groups. Taken together, the population structure analyses consistently demonstrated that Tibetan pigs formed a distinct genetic cluster, separate from wild boars. At the same time, Tibetan pigs were not genetically homogeneous, indicating that the four Tibetan pig groups have likely experienced different demographic histories.

### 3.3. Demographic History of the Four Tibetan Pig Groups

**Figure 2:**
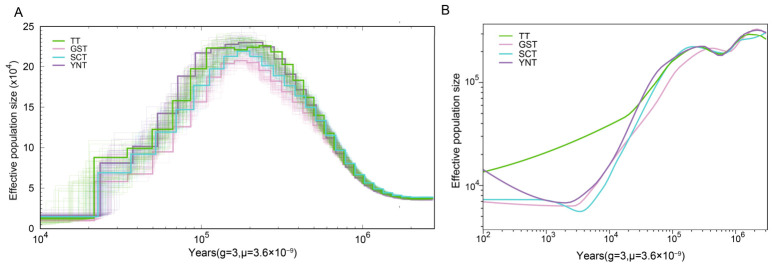
Demographic history of the four Tibetan pig groups inferred by PSMC and SMC++. (**A**) Historical effective population size (Ne) inferred by PSMC using one high-coverage individual from each Tibetan pig group with 80 bootstraps. (**B**) Ne inferred by SMC++ using genotype data from all individuals in each Tibetan pig group. TT, Tibetan pigs from Tibet; GST, Tibetan pigs from Gansu; YNT, Tibetan pigs from Yunnan; SCT, Tibetan pigs from Sichuan.

PSMC analysis was performed using one high-coverage individual from each Tibetan pig group. The results showed that the four Tibetan pig groups shared broadly similar trajectories of historical effective population size (Ne) across deep timescales (Figure 2A). This pattern suggested that Tibetan pigs from different regions may have originated from a limited number of ancestral founders. All four Tibetan pig groups exhibited an evident decline in Ne at approximately 200 Kya (kilo years ago), indicating a shared historical bottleneck. Notably, GST consistently showed the smallest effective population size across the reconstructed timescale, which was consistent with the results of nucleotide diversity and LD decay analyses.

Because PSMC infers historical population size from the distribution of heterozygous sites along the genome using a single diploid genome, it is mainly informative for deep demographic history, but is less sensitive to recent changes and is limited by the small number of individuals that can be analyzed. To better resolve recent demographic changes, we further applied SMC++ using genotype data from all individuals (Figure 2B). The SMC++ results also showed that GST had the smallest effective population size among the four Tibetan pig groups, supporting a long-term reduction in population size in this group. In addition, TT began to show a distinct demographic trajectory relative to the other three Tibetan pig groups at approximately 30 Kya under the mutation rate and generation time assumptions used here. Because no explicit sensitivity analyses were performed for these scaling parameters, this timescale should be interpreted cautiously. By contrast, GST, YNT, and SCT showed highly similar Ne trajectories across most of the recent timescale, indicating that these three groups shared a more similar recent demographic history. Notably, YNT showed a moderate increase in Ne at around 2 Kya, which may reflect a recent population expansion or external genetic input. Taken together, the demographic analyses suggest that the four Tibetan pig groups share a common deep ancestral background, but have become differentiated in their recent demographic histories. Among them, TT showed a distinct recent demographic trajectory relative to the other three Tibetan pig groups, whereas GST consistently maintained the smallest effective population size.

### 3.4. The Origins of TT

**Figure 3:**
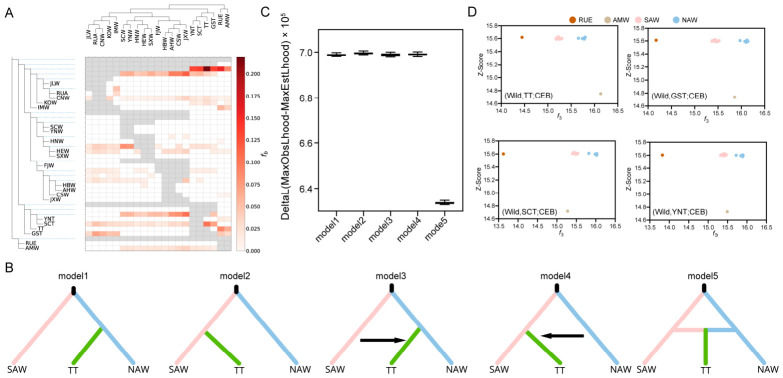
The analysis of TT origin. (**A**) Heat map of the f-branch metric for wild boars and Tibetan pigs. (**B**) Schematic illustration of the five demographic models tested for the origin of TT using fastsimcoal2 based on multidimensional SFS. Model 1: TT split from NAW after the divergence of SAW and NAW. Model 2: TT split from SAW after the divergence of SAW and NAW. Model 3: TT split from NAW and subsequently received gene flow from SAW. Model 4: TT split from SAW and subsequently received gene flow from NAW. Model 5: TT originated through admixture between SAW and NAW. (**C**) Comparison of the five demographic models based on delta-likelihood values, showing that model 5 provided the best fit to the data. (**D**) Outgroup f3 statistics of the form f3(wild boar, Tibetan pig; CEB). CEB, *Sus cebifrons*; TT, Tibetan pigs from Tibet; GST, Tibetan pigs from Gansu; YNT, Tibetan pigs from Yunnan; SCT, Tibetan pigs from Sichuan; SAW, southern Asian wild boars; NAW, northern Asian wild boars.

To further investigate the origin of Tibetan pigs, we first used the f-branch approach to evaluate excess allele sharing between TBT and different wild boar groups (Figure 3A). At the level of the branch representing the four Tibetan pig groups as a whole, no strong signal toward a single external lineage was detected. We found that different Tibetan pig groups showed different affinities, with GST being more closely associated with northern-related wild boars, whereas YNT/SCT showed stronger affinity to southern-related groups. In contrast, the terminal branch of TT showed a much more distinct pattern, with clearly elevated f-branch values for RUE and AMW relative to most other wild boar groups.

Given that TT represented the earliest segregating trajectory in the demographic analysis, we further analyzed the multidimensional site frequency spectra (SFS) using the composite likelihood method. (Figure 3B). We tested five models to simulate the demographic history of Tibetan pigs: model 1, TT split from NAW after the divergence of SAW and NAW; model 2, TT split from SAW after the divergence of SAW and NAW; model 3, TT split from NAW and subsequently received gene flow from SAW; model 4, TT split from SAW and subsequently received gene flow from NAW; and model 5, TT originated through admixture between SAW and NAW. These models represent simplified competing demographic scenarios rather than an exhaustive representation of all possible domestication and gene-flow histories. The model comparison showed that model 5 clearly provided the best fit among the five tested models, as indicated by the lowest delta-likelihood values by 100 replicates (Figure 3C and Supplementary Table S4). This ranking was further supported by Akaike information criterion (AIC) based model comparison (Supplementary Table S3). Under the tested demographic models, TT was best supported by a mixed-origin scenario involving both SAW- and NAW-related ancestral components. To evaluate the robustness of this inference, we further repeated the analyses using a folded SFS and a randomly down-sampled dataset. In the folded-SFS analysis, the relative fit of model 3 and model 4 improved, but model 5 remained the best-fitting model (Supplementary Figure S4A). Similarly, in the down-sampled analysis, model 3 showed improved fit relative to model 1, model 2, and model 4, but model 5 still remained the best-supported model (Supplementary Figure S4B). These sensitivity analyses indicate that ancestral-state assignment and sample size affect the relative fit of some alternative models, but do not change the overall support for model 5 as the best-fitting model for TT.

To further assess the overall genetic affinity between TT and different wild boar groups, we calculated outgroup *f*_3_ statistics (Figure 3D). The results showed that all four Tibetan pig groups shared more genetic drift with NAW than with SAW, indicating that northern-related ancestry represents an important component in Tibetan pigs. Notably, TT showed an elevated *f*_3_ value with AMW, suggesting substantial genome-wide drift sharing between TT and AMW. When considered together with the f-branch results, this pattern indicates that, although the best-fitting demographic model for TT is a SAW-NAW admixture model, TT still retains a detectable WEW-related excess sharing signal.

### 3.5. The Origin of the Other Tibetan Pig Groups

**Figure 4:**
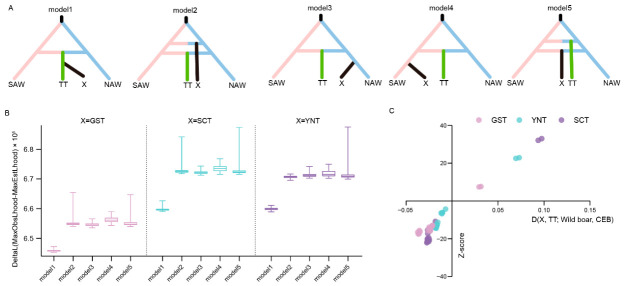
Demographic modeling supports GST, YNT, and SCT as lineages diverging after the formation of the TT-related lineage under the tested models. (**A**) Schematic illustration of the five demographic models tested for the origins of GST, YNT, and SCT under the mixed-origin framework of TT. In model 1, X (GST, YNT, or SCT) splits from TT after the admixed origin of TT. In model 2, X also had an admixed origin, and the admixture event occurred earlier than that of TT. In model 3, X splits independently from NAW after the admixed origin of TT. In model 4, X splits independently from SAW after the admixed origin of TT. In model 5, X also had an admixed origin, but the admixture event occurred later than that of TT. (**B**) Comparison of the five demographic models based on delta-likelihood values from 100 independent fastsimcoal2 runs for each Tibetan pig group. Model 1 consistently showed the best fit for GST, YNT, and SCT. (**C**) D-statistics of the form D (X, TT; wild boar, CEB). CEB, *Sus cebifrons*; TT, Tibetan pigs from Tibet; GST, Tibetan pigs from Gansu; YNT, Tibetan pigs from Yunnan; SCT, Tibetan pigs from Sichuan; SAW, southern Asian wild boars; NAW, northern Asian wild boars.

After identifying TT as best explained by an admixture model involving SAW- and NAW-related ancestral sources, we further investigated the possible origins of the other three Tibetan pig groups. To address this question, five alternative demographic models were tested (Figure 4A). In model 1, X (GST, YNT, or SCT) splits from TT after the admixed origin of TT. In model 2, X also had an admixed origin, and the admixture event occurred earlier than that of TT. In model 3, X splits independently from NAW after the admixed origin of TT. In model 4, X splits independently from SAW after the admixed origin of TT. In model 5, X also had an admixed origin, but the admixture event occurred later than that of TT. These models were designed to distinguish whether the other Tibetan pig groups originated as descendant lineages of TT or instead represented independent lineages derived directly from NAW, SAW, or their admixture. The fastsimcoal2 simulations based on the multidimensional SFS consistently showed that model 1 provided the best fit for GST, YNT, and SCT, based on 100 independent runs for each group (Figure 4B). Compared with the other four models, model 1 showed the highest overall delta-likelihood values and the most stable pattern across replicates. This ranking was further supported by an AIC-based model comparison (Supplementary Table S3). Under the tested demographic models, this result supports a scenario in which GST, YNT, and SCT were more likely to have diverged after the formation of the TT-related lineage, rather than representing independent origins from NAW, SAW, or separate admixture events. To assess the robustness of this inference, we further repeated the analyses using folded SFS and randomly down-sampled datasets. In the folded-SFS analysis, the fit of model 2 improved, but model 1 still remained the best-fitting model for GST, YNT, and SCT (Supplementary Figure S5A). In the down-sampled analysis, the fit of model 2 and model 4 improved to some extent, but model 1 still showed the best fit overall (Supplementary Figure S5B). These sensitivity analyses suggest that sample size and ancestral-state polarization do influence the relative fit among competing models, but the overall support for model 1 remained unchanged.

To further evaluate the relative genetic affinities between TT and the other Tibetan pig groups, we performed D-statistics of the form D (X, TT; wild boar, CEB) (Figure 4C and Supplementary Table S5). The results showed that, relative to TT, GST, YNT, and SCT shared more alleles with both SAW and NAW, whereas TT shared more alleles with AMW and RUE. This pattern indicates that, although demographic modelling supports GST, YNT, and SCT as lineages derived from TT, these three groups retained more allele sharing with East Asian wild boars overall, while TT showed stronger genetic affinity to western Eurasian wild boars. Together with the results presented above, this suggests that TT experienced a more complex demographic history and accumulated stronger WEW-related signals than the other Tibetan pig groups. By contrast, GST, YNT, and SCT, although derived from TT, appear to have remained genetically closer to the East Asian wild boar background during their subsequent evolution.

### 3.6. Western Eurasian-Related Excess-Sharing Signal in TT

**Figure 5:**
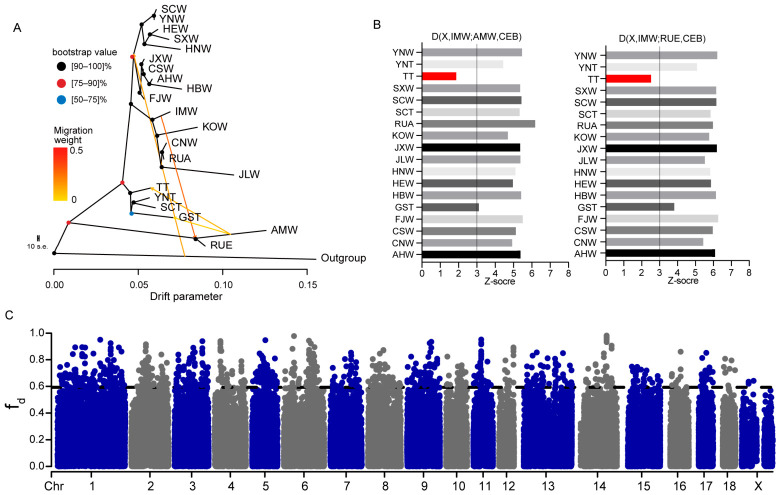
Western Eurasian-related excess-sharing signal in TT. (**A**) TreeMix analysis with M = 4. The consensus tree was constructed from an ML tree using 500 bootstrap replicates, and the bootstrap support values for each node are indicated by different colors. (**B**) Z-scores of D-statistics in the form D (X, IMW; [AMW, RUE], CEB), used to evaluate whether the genetic affinity between TT and WEW-related wild boars could be explained through IMW. (**C**) Genome-wide sliding-window f_d_ analysis for detecting candidate excess-sharing windows associated with AMW-related ancestry in TT. The horizontal dashed line indicates the top 1% threshold of the f_d_ distribution.

In the ADMIXTURE analysis, we observed that TT consistently retained a WEW-related ancestry component, regardless of the number of K tested (Supplementary Figure S3). In addition, D-statistics showed that, compared with the other three Tibetan pig groups, TT consistently shared more genetic drift with AMW (Figure 4C). Together, these results suggested the presence of an additional western Eurasian-related signal in TT. To further investigate this pattern, we used TreeMix to evaluate whether migration edges involving TT were repeatedly recovered across models with different numbers of migration edges. When the number of migration edges was set to M = 1, a migration edge from WEW to TT was detected. Importantly, this signal remained stable as the number of migration edges increased, indicating that this pattern was consistently recovered across models with increasing migration edges (Supplementary Figure S6). When M = 2, we also detected a migration edge between *S. cebifrons* and wild boars from Fujian, which was consistent with previous interpretations of wild boar dispersal routes from the Fujian region toward Southeast Asian islands mediated by Austronesian [37]. The OptM result showed that M = 4 had the strongest support (Supplementary Figure S7). When M = 4, an additional introgression event from IMW to WEW was identified (Figure 5A). Because IMW also showed genetic affinity with WEW, we further tested whether the inferred gene flow from AMW to TT might have been mediated through IMW. To address this question, we calculated D-statistics of the form D (X, IMW; WEW [AMW, RUE], CEB) to examine the relative genetic relationships among IMW, TT, AMW, and RUE (Figure 5B). The results showed that, compared with the other wild boar or Tibetan pig groups, IMW exhibited significantly increased genetic affinity with WEW (|Z| > 3). However, when X = TT, the difference between IMW and TT in their affinity to WEW was not significant (|Z| < 3). These results do not support the hypothesis that the WEW-related signal in TT was mainly mediated through IMW.

To further compare whether the putative introgressed genomic regions were similar between the AMW-IMW and AMW-TT pairs, we performed genome-wide sliding-window f_d_ analyses based on D (NAW, IMW; AMW, CEB) and D (TT, GST; AMW, CEB). After selecting the top 1% f_d_ windows as candidate excess-sharing windows and taking their intersection, we found that the overlap between the candidate AMW-related windows in the AMW-IMW and AMW-TT comparisons accounted for only 10.4% of the candidate AMW-related windows identified in TT (Supplementary Tables S6 and S7). This result further indicates that the AMW-related excess-sharing pattern observed in TT is not highly similar to that between AMW and IMW, and therefore cannot support the hypothesis that the WEW-related component in TT was mainly introduced through IMW. Within the top 1% candidate AMW-related windows in TT, we identified several biologically plausible candidate genes associated with pulmonary vascular response under hypoxia or susceptibility to pulmonary hypertension, including *BMPR2* [38], *JAK2* [39], *IL33* [40], and *MIF* [41] (Figure 5C). These genes represent biologically plausible candidates that may be relevant to hypoxia-related adaptation in TT.

Topology weighting analysis using Twisst2 further showed that the topology (((AMW, TT), SAW), CEB) had a higher genome-wide weight than (((AMW, SAW), TT), CEB), supporting excess AMW-TT sharing, although incomplete lineage sorting and ancient shared variation cannot be fully excluded (Supplementary Figure S8).

### 3.7. Genome-Wide Selective Sweep in Tibetan Pigs

**Figure 6:**
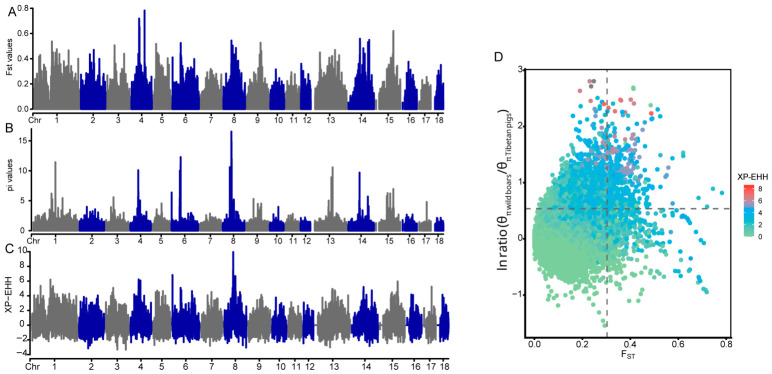
Genome-wide selective sweep in Tibetan pigs. (**A**) Manhattan plot of genome-wide Fst values between Tibetan pigs and wild boars. (**B**) Manhattan plot of θπ ratio values, calculated as π (wild boars)/π (Tibetan pigs). (**C**) Manhattan plot of genome-wide XP-EHH values between Tibetan pigs and wild boars. (**D**) Distribution of the Fst (x axis), π ln ratio (y axis), and XP-EHH value (colored) between wild boars and Tibetan pigs.

To identify potential candidate genes associated with high-altitude adaptation in Tibetan pigs, we first calculated Fst values between the four Tibetan pig groups and the Asian wild boar groups. Using the top 5% of windows (Fst > 0.180465) as the cutoff, we identified 5810 candidate genomic regions (Figure 6A). We then screened genomic regions showing reduced genetic diversity in Tibetan pigs using π (wild boars)/π (Tibetan pigs), followed by log10 standardization of the ratio (Figure 6B). We further performed a whole-genome scan using the haplotype-based XP-EHH method, and using the top 5% as the cutoff, a total of 6260 genomic regions (XP-EHH > 1.7421) were identified (Figure 6C). By intersecting the results from the three methods, we ultimately identified 692 non-redundant candidate genes, suggesting that these genes may have experienced strong selection during the adaptation of Tibetan pigs to high-altitude environments (Figure 6D).

QTL enrichment analysis of selective sweep regions identified several trait categories potentially related to high-altitude adaptation in Tibetan pigs. Among them, the most notable signals were associated with hematological traits, including red blood cell count, mean corpuscular volume, mean corpuscular hemoglobin content, and mean corpuscular hemoglobin concentration. These traits are closely related to blood oxygen transport and therefore may be relevant to adaptation to hypoxic environments. In addition, platelet count and mean platelet volume were also enriched, suggesting potential roles of the selected regions in platelet function and blood circulation under hypoxic stress (Supplementary Figure S9). Further GO enrichment analysis showed that the candidate genes were significantly enriched in several biological processes related to environmental adaptation, including cellular response to UV (*BMF*, *NOC2L*, *TMEM161A*, *ATR*, *DHX36*, *CREBBP*), regulation of systemic arterial blood pressure (*ADM5*, *GSK3A*, *FSHR*), positive regulation of vascular endothelial cell proliferation (*PDPK1*, *SIRT6*), and lung alveolus development (*CIC*, *PHF14*, *PGR*) (Supplementary Table S8). These enrichment results suggest that the selected genes may be involved in the development and functional regulation of organs closely related to high-altitude adaptation, particularly the heart, blood vessels, and lung tissues. In addition, we identified several important candidate genes that have previously been reported to be directly associated with hypoxia adaptation or with alleviating hypoxic damage, including *EGLN2* [42], *HIF3A* [43], *ADORA2A* [44], and *ADCY9* [45]. These genes may play key roles in the adaptation of Tibetan pigs to hypoxic conditions at high altitude.

## 4. Discussion

Our results supported the view that Tibetan pigs should not be treated as a single homogeneous high-altitude population, because the four Tibetan pig groups differed in genetic structure, demographic history, and ancestry composition, which is broadly consistent with previous studies showing substantial regional differentiation among Tibetan pigs [8,46]. Demographic history simulation based on multidimensional SFS revealed that TT appeared to occupy a particularly important position, because it was best explained by a mixed-origin model involving both NAW- and SAW-related ancestral components, whereas GST, YNT, and SCT were better fitted as lineages derived after the formation of TT. Such a scenario is biologically plausible in light of archaeological evidence indicating that domestic pigs reached the Tibetan Plateau together with millet-based farming systems, and that pig husbandry expanded into high-elevation regions as part of broader movements of people, crops, and livestock [47]. The outgroup *f*_3_ results showed that TT still shared substantial drift with NAW, indicating that a northern-related ancestral component remained an important part of its genomic background, which is consistent with previous results using mitochondrial data that found that Tibetan pigs originated from the upstream region of the Yellow River [48]. The fact that GST, YNT, and SCT were better modeled as descendants of TT than as independent derivatives of NAW, SAW, or separate admixture events further suggests that TT may represent a key ancestral branch in the formation of Tibetan pigs. However, GST retained stronger NAW-related affinity, whereas YNT and SCT were relatively closer to SAW-related backgrounds, implying that subsequent divergence from TT was followed by region-specific demographic processes. This interpretation is in line with previous work showing that Tibetan pig populations were shaped not only by shared plateau ancestry but also by admixture with geographically neighboring lowland populations [7].

The PSMC and SMC++ results should also be interpreted in the context of their different methodological properties. PSMC is based on a single diploid genome and is mainly informative for broad long-term demographic trends, whereas SMC++ can incorporate multiple unphased individuals and therefore has higher resolution for more recent demographic history [28]. In this study, the broadly similar PSMC trajectories of the four Tibetan pig groups likely reflect their shared deep ancestral background, whereas the SMC++ results more clearly captured the recent differentiation of TT from the other three groups. Rather than indicating an exact divergence time, the estimated point around 30 Kya is better interpreted as the approximate time at which TT began to show a demographic trajectory distinct from those of GST, YNT, and SCT under the scaling assumptions used here. The four Tibetan pig groups were not genetically homogeneous, but instead showed clear differences in genetic diversity, LD decay, and effective population size. In particular, GST exhibited the lowest nucleotide diversity, the slowest LD decay among Tibetan pig groups, and the smallest effective population size, suggesting that this population may have experienced stronger drift or a more restricted demographic history.

The WEW-related ancestry component in ADMIXTURE, the excess allele sharing between TT and AMW/RUE in D-statics and f-branch analyses, and the repeatedly recovered TreeMix migration edge connecting WEW and TT all indicate that TT retained a stronger western Eurasian-related signal than the other Tibetan pig groups. However, this pattern should not be over-interpreted as proof of direct historical migration specifically from modern Armenian wild boars into TT [49]. As emphasized in recent discussions of introgression tests, sampled populations can act as proxies for unsampled or extinct donor lineages, and incomplete sampling may bias the apparent source of gene flow [49]. For that reason, AMW is best interpreted here as the closest sampled representative of a western Eurasian-related ancestry component, rather than the only possible donor population. The limited overlap between the top introgressed windows in the AMW-IMW and AMW-TT comparisons also supports the view that the western Eurasian-related component in TT was not mainly transmitted through IMW alone. This result is noteworthy because long-distance introgression has also been reported in other domestic animals from high-altitude systems [14,50,51]. In Tibetan sheep, for example, adaptive introgression from argali has been shown to contribute to plateau adaptation [14]. Although pigs and sheep clearly differ in domestication history and dispersal routes, these studies show that introgression from geographically distant lineages can contribute to the genomic architecture of high-altitude livestock. Among the top AMW-related candidate regions in TT, *BMPR2*, *JAK2*, *IL33*, and *MIF* are especially notable because all three genes are functionally linked to pulmonary vascular biology and hypoxia-related pathology [38,39,40,41,52]. These functional links make it plausible that introgression from wild boars of the Caucasus region may have contributed variants relevant to pulmonary vascular response.

The results of selective sweeps also pointed to some genes that may contribute to the adaptability of Tibetan pigs to plateaus. Genes such as *EGLN2*, *HIF3A*, *ADORA2A*, and *ADCY9* all fit biological processes that are relevant to hypoxia sensing, lung development, vascular regulation, or cardiovascular signaling. *EGLN2* belongs to the core EGLN-HIF oxygen-sensing system, which is central to cellular responses to oxygen availability across vertebrates [53]. *HIF3A* is less studied than other HIF family members, but available evidence indicates that it is responsive to hypoxia and contributes to lung development and alveolarization [54]. *ADORA2A* is part of the hypoxia-responsive adenosine signaling network and has been linked to endothelial metabolic responses and vascular regulation under low-oxygen conditions [44]. *ADCY9* is reported to be potentially related to plateau adaptation in Peruvian populations [45]. In addition to these candidate genes, the QTL enrichment results provide further support for the biological relevance of the selected regions. Notably, multiple enriched QTLs were associated with hematological traits, including red blood cell count, mean corpuscular volume, mean corpuscular hemoglobin content, mean corpuscular hemoglobin concentration, platelet count, and mean platelet volume. These traits are directly or indirectly related to oxygen transport capacity, blood rheology, and circulatory regulation, all of which are highly relevant to adaptation in hypoxic high-altitude environments.

Several limitations of the present study should be acknowledged. First, although our dataset included Tibetan pigs from four regions, the sample sizes of these groups remained limited and uneven, which may affect the precision of demographic inference. Second, the fastsimcoal2 analyses were performed within a predefined set of candidate models, and the best-supported model should therefore be interpreted as the best fit among the models tested here rather than as the uniquely true demographic history of Tibetan pigs. Third, the unfolded SFS relied on ancestral-state inference based on a limited number of *S. cebifrons* individuals, which may introduce uncertainty into demographic modeling. Although the folded-SFS analyses supported the same best-fitting models, they do not fully eliminate the possibility of residual bias caused by limited outgroup-based polarization. Fourth, many analyses support excess western Eurasian-related sharing in TT, but these approaches cannot by themselves fully distinguish direct directional introgression from incomplete lineage sorting, ancient shared variation, or unsampled ghost populations. In addition, migration edges in our TreeMix analyses did not have direct bootstrap support and should therefore be interpreted cautiously. Finally, the selective sweep and candidate gene analyses remain exploratory, and the biological roles of these genes in high-altitude adaptation will require further validation through functional studies. Future work incorporating denser sampling from more Tibetan pigs and lowland domestic pigs will be important for refining the demographic and introgression history of Tibetan pigs.

## 5. Conclusions

In conclusion, our results show that Tibetan pigs are not genetically homogeneous and that the Tibetan pig population from TT has a demographic history distinct from that of the other three Tibetan pig groups. Under the demographic models tested in this study, TT was best supported by a mixed-origin scenario involving both NAW- and SAW-related ancestry, whereas GST, YNT, and SCT were best supported as lineages diverging after the formation of the TT-related lineage. In addition, TT retained a potential western Eurasian-related signal, consistent with excess allele sharing or introgression, although direct gene flow from a specific sampled population cannot yet be established. Together, these findings provide a working hierarchical framework for understanding the origin and differentiation of Tibetan pigs under the demographic models tested here. Future studies incorporating denser sampling, additional lowland domestic pigs and Tibetan pigs, and functional validation of candidate genes will be important for refining this framework.

## Data Availability

The accession IDs of the raw data are available in Supplementary Table S1.

## Supplementary Materials

The following supporting information can be downloaded at: https://www.mdpi.com/article/10.3390/ani16091328/s1, Figure S1: Photos of Tibetan pigs from four regions. The photo is cited from the second edition of *ANIMIAL GENETIC RESOURCES IN CHINA: PIGS*; Figure S2: Cross-validation error in admixture analysis with different K values; Figure S3: Population genetic structure of global pigs inferred from ADMIXTURE (K = 2–9) with species names labeled with abbreviated names; Figure S4: Sensitivity analysis for origin of TT; Figure S5: Sensitivity analysis for origin of other Tibetan pigs; Figure S6: TreeMix analysis revealed migration events of wild boars and Tibetan pigs (m = 2–7); Figure S7: The result of OptM analysis; Figure S8: Topology weighting analysis of the four groups; Figure S9: QTL enrichment analysis of selective sweep regions; Table S1: Sample information for analysis in this study; Table S2: The annotation of the SNPs; Table S3: Akaike information criterion results of all models tested in this study; Table S4: Inferred parameters of demographic history of TT; Table S5: Allele sharing between wild boars and Tibetan pigs; Table S6: Top 1% introgression regions calculated using the D (GST, TT; AMW, CEB); Table S7: Top 1% introgression regions calculated using the D (NAW, IMW; AMW, CEB); Table S8: The GO enrichment analysis of selected genes.

## Abbreviations

The following abbreviations are used in this manuscript:

SAW	southern Asian wild boars
NAW	northern Asian wild boars
TT	Tibetan pigs from Tibet
GST	Tibetan pigs from Gansu province
YNT	Tibetan pigs from Yunnan province
SCT	Tibetan pigs from Sichuan province
SFS	site frequency spectrum
Ne	effective population size
