# Supplementary material for: Genomics Insights into the Demographic History and Introgression of Tibetan Pigs
Source: Animals (Basel). 2026 Apr 27;16(9):1328. doi: 10.3390/ani16091328 (PMC13162695; doi:10.3390/ani16091328)
Supplement: Supplementary file 1 [file animals-16-01328-s001.zip › Supplementary Figure.pdf]

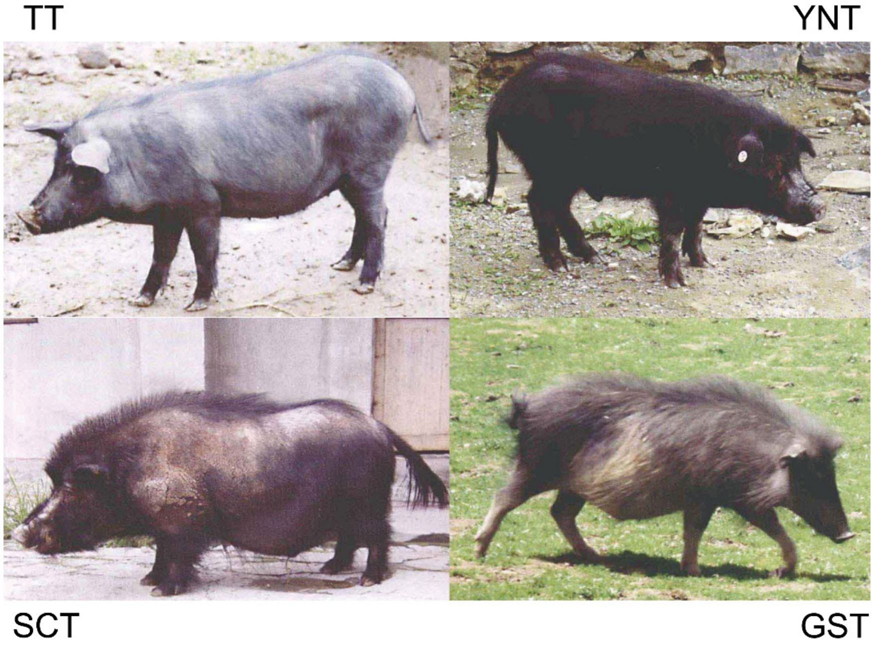

Supplementary Figure S1. Photos of Tibetan pigs from four regions. The photo is cited from the second edition of *ANIMIAL GENETIC RESOURCES IN CHINA: PIGS*

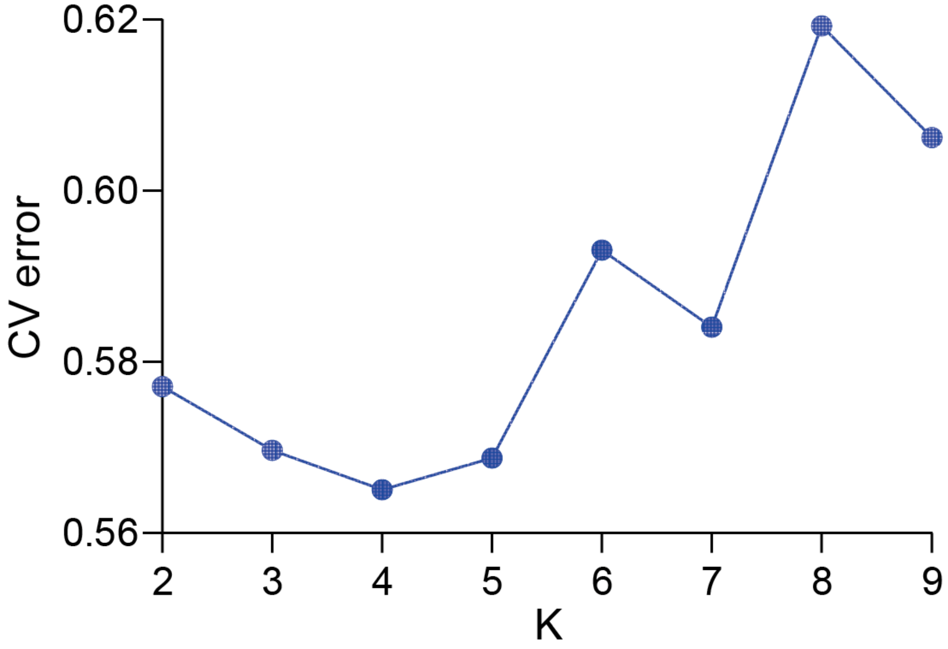

Supplementary Figure S2. Cross-validation error in admixture analysis with different K values.

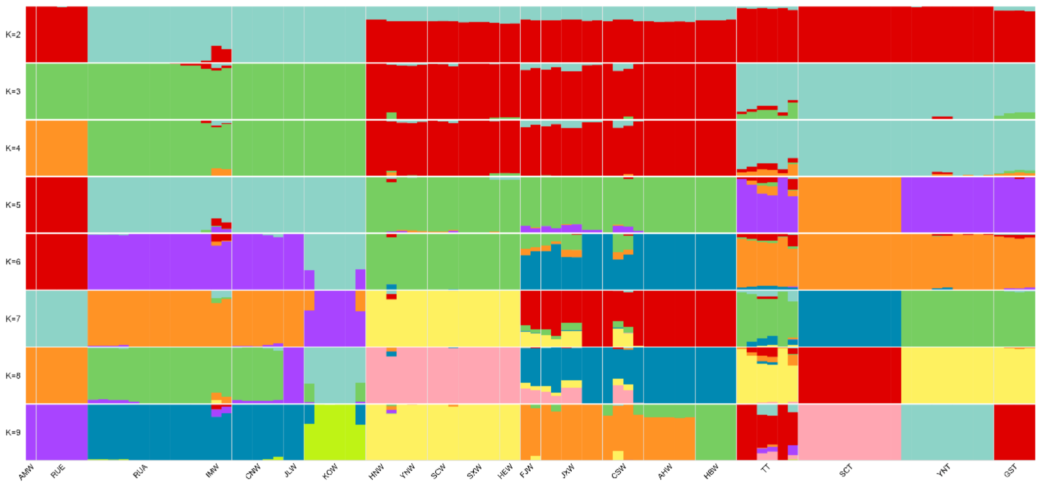

Supplementary Figure S3. Population genetic structure of global pigs inferred from ADMIXTURE (K=2-9) with species names labeled with abbreviated names.

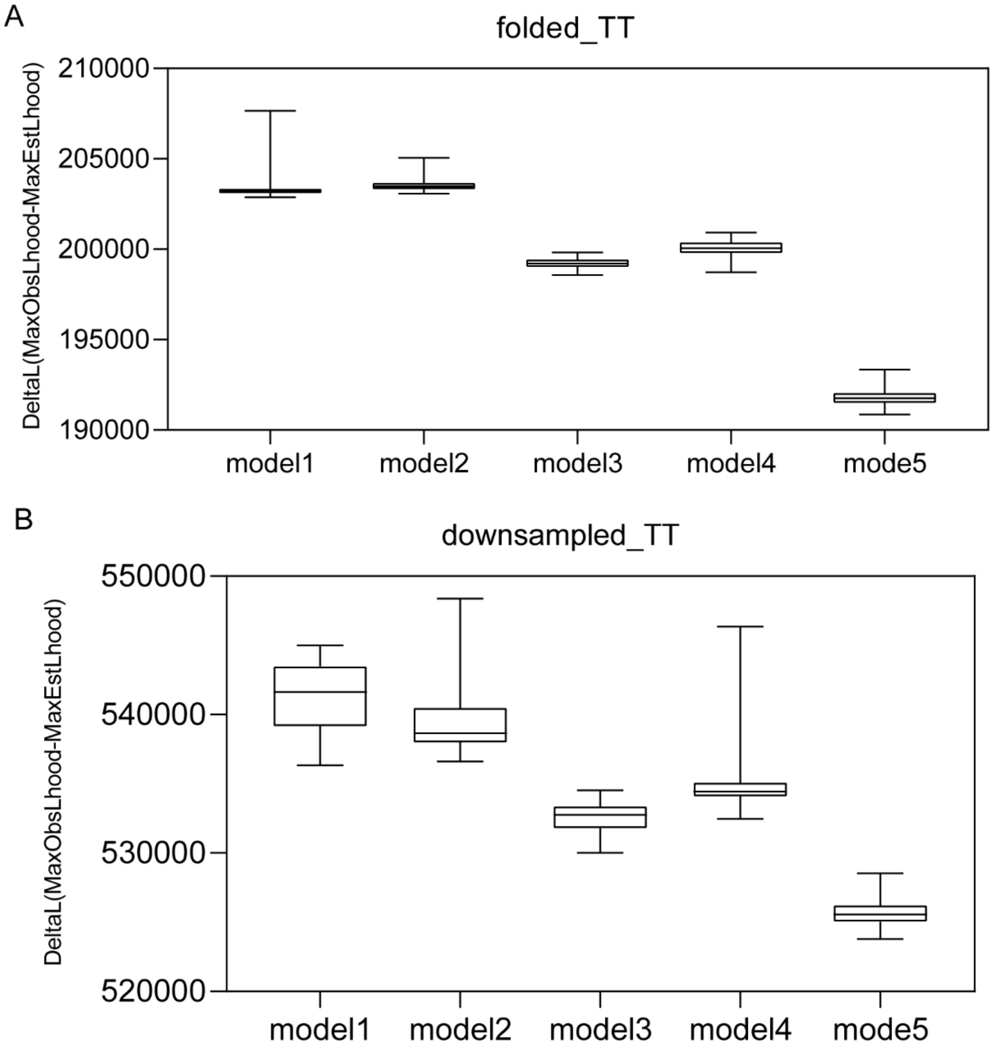

Supplementary Figure S4. Sensitivity analysis for origin of TT.

(A) Comparison of the five demographic models based on delta-likelihood values using folded SFS. (B) Comparison of the five demographic models based on delta-likelihood values using unfolded SFS after down sample.

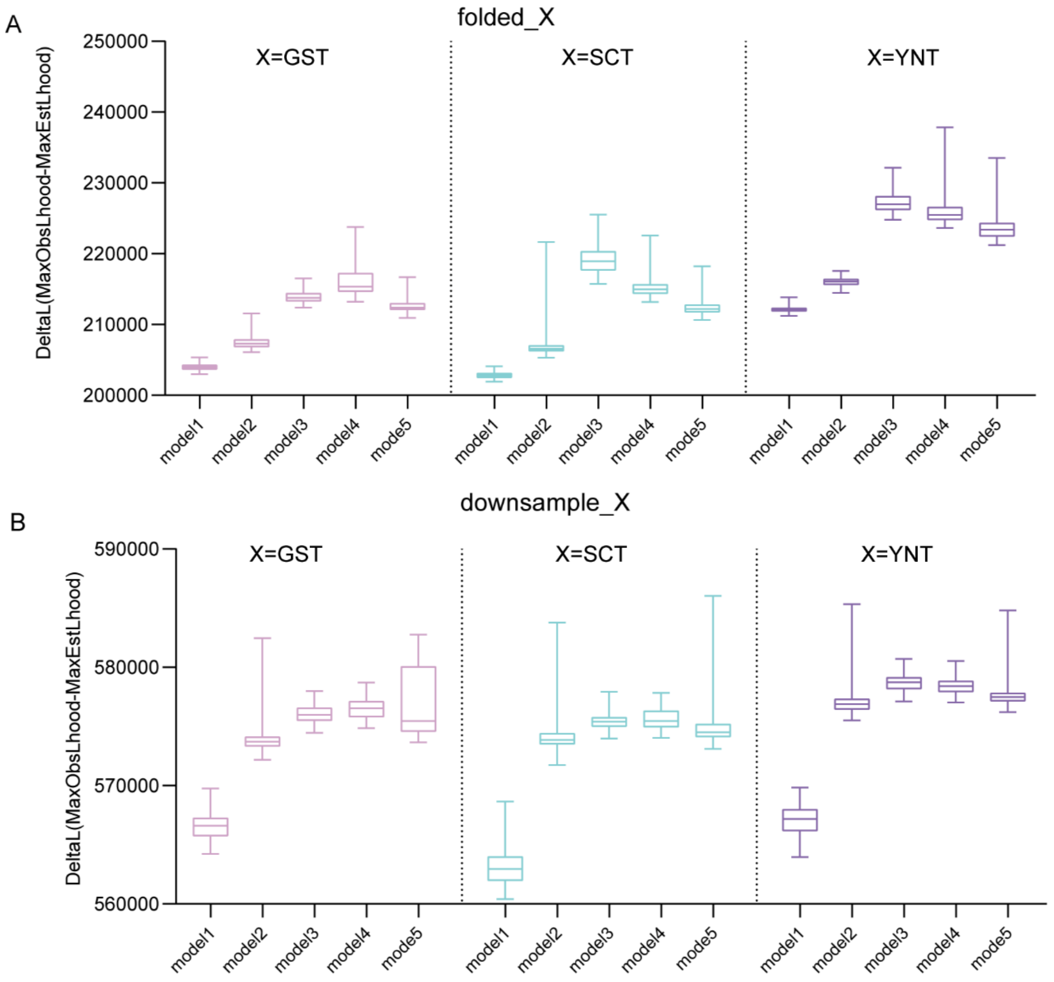

Supplementary Figure S5. Sensitivity analysis for origin of other Tibetan pigs.

(A) Comparison of the five demographic models based on delta-likelihood values using folded SFS. (B) Comparison of the five demographic models based on delta-likelihood values using unfolded SFS after down sample.

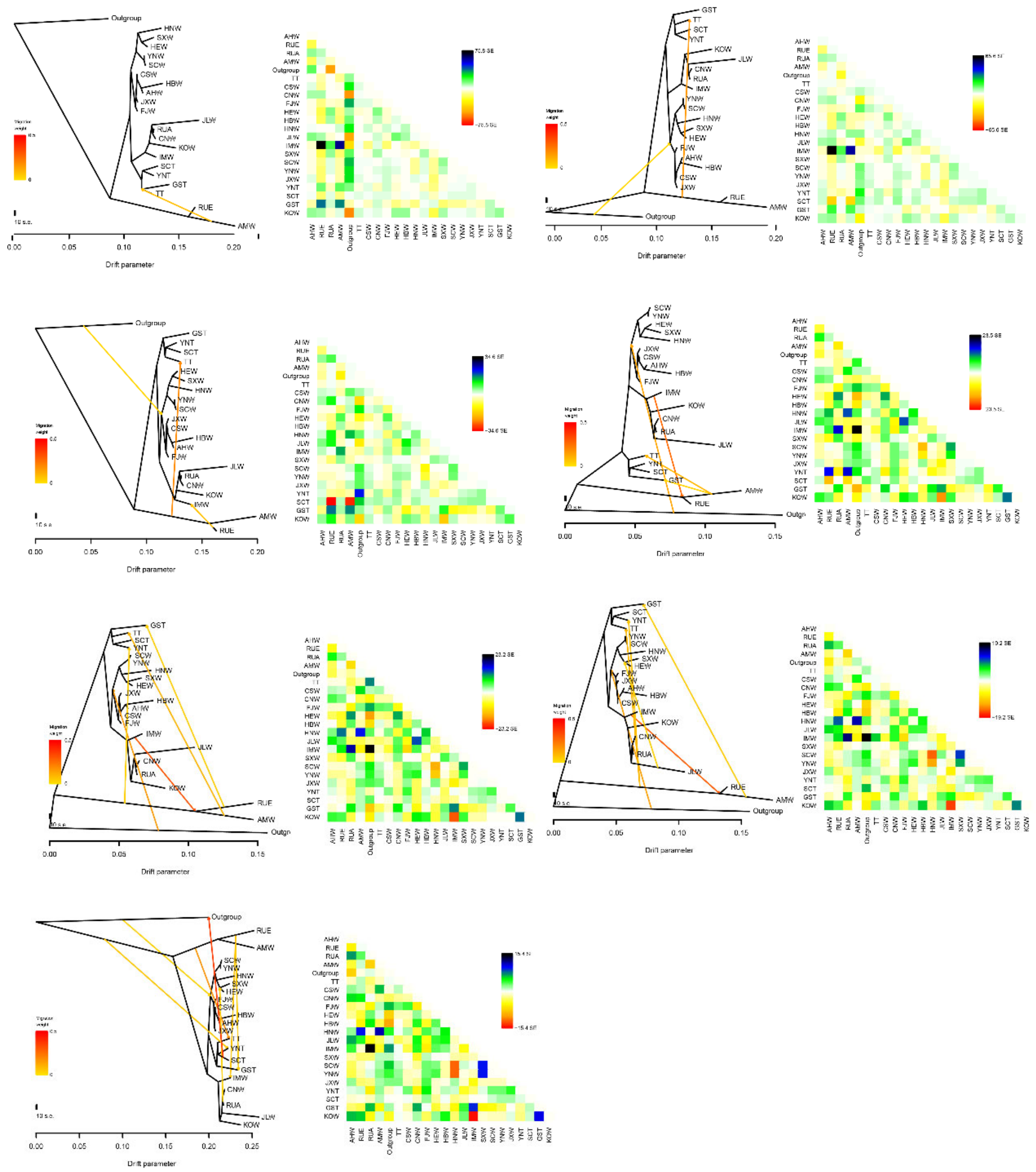

Supplementary Figure S6. TreeMix analysis revealed migration events of wild boars and Tibetan pigs (m = 2-7)

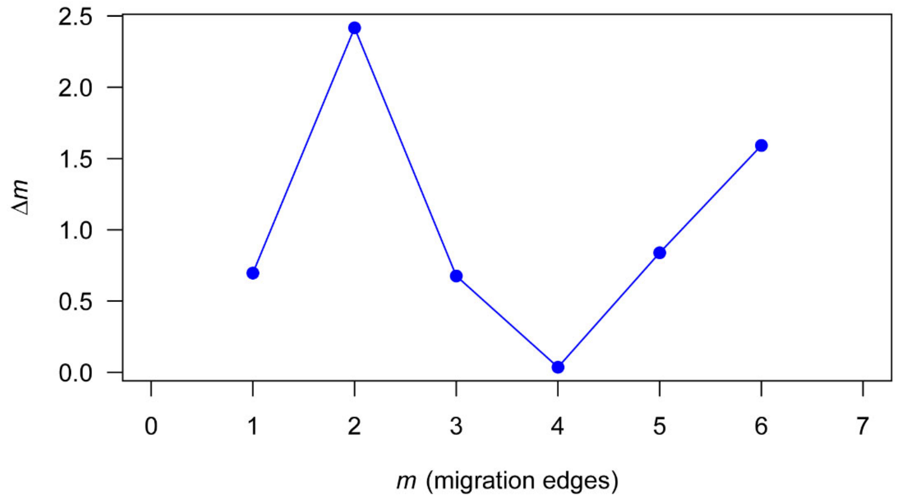

Supplementary Figure S7. The result of OptM analysis.

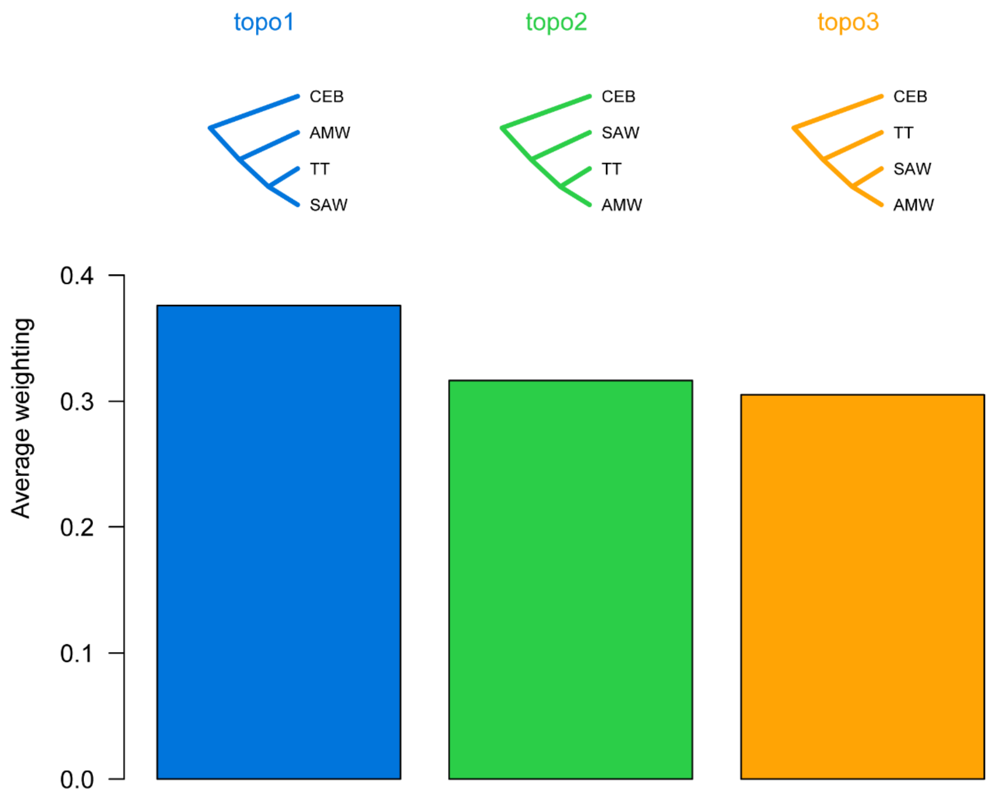

Supplementary Fig. 8 Topology weighting analysis of the four groups.

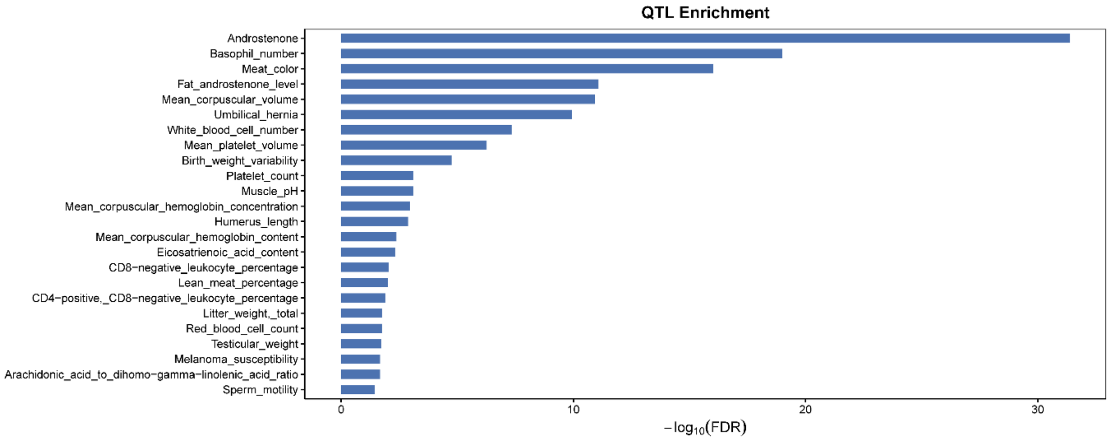

Supplementary Fig. 9 QTL enrichment analysis of selective sweep regions.
